# Control of Relative Air Humidity as a Potential Means to Improve Hygiene on Surfaces: A Preliminary Approach with *Listeria monocytogenes*

**DOI:** 10.1371/journal.pone.0148418

**Published:** 2016-02-03

**Authors:** Fiona Zoz, Cyril Iaconelli, Emilie Lang, Hayet Iddir, Stéphane Guyot, Cosette Grandvalet, Patrick Gervais, Laurent Beney

**Affiliations:** UMR PAM Université Bourgogne-Franche-Comté / AgroSup Dijon, Dijon, France; University of Aveiro, PORTUGAL

## Abstract

Relative air humidity fluctuations could potentially affect the development and persistence of pathogenic microorganisms in their environments. This study aimed to characterize the impact of relative air humidity (RH) variations on the survival of *Listeria monocytogenes*, a bacterium persisting on food processing plant surfaces. To assess conditions leading to the lowest survival rate, four strains of *L*. *monocytogenes* (EGDe, CCL500, CCL128, and LO28) were exposed to different RH conditions (75%, 68%, 43% and 11%) with different drying kinetics and then rehydrated either progressively or instantaneously. The main factors that affected the survival of *L*. *monocytogenes* were RH level and rehydration kinetics. Lowest survival rates between 1% and 0.001% were obtained after 3 hours of treatment under optimal conditions (68% RH and instantaneous rehydration). The survival rate was decreased under 0.001% after prolonged exposure (16h) of cells under optimal conditions. Application of two successive dehydration and rehydration cycles led to an additional decrease in survival rate. This preliminary study, performed in model conditions with *L*. *monocytogenes*, showed that controlled ambient RH fluctuations could offer new possibilities to control foodborne pathogens in food processing environments and improve food safety.

## Introduction

*Listeria monocytogenes*, the causative agent of listeriosis, is an ubiquitous Gram-positive bacterium distributed in many environments [1–4] and is also frequently isolated in food processing environments or in raw food products [5–9]. *Listeria monocytogenes* primarily enters the food-production chain by cross-contamination from surfaces and equipment, making this pathogen a major threat to the food industry and consumers [10–12]. Human listeriosis occurs only occasionally, but potentially causes severe infection in vulnerable people, such as pregnant women, unborn infants, neonates, the elderly, and immunocompromised people, and might cause febrile gastroenteritis in healthy people [13, 14]. Furthermore, product contamination leads to a significant economic impact on food industry due to recall and destruction of incriminated products. Like most other microorganisms, *L*. *monocytogenes* is adapted to many otherwise stressful environmental conditions as reviewed by Gandhi and Chikindas (2007) [15]. It can grow in high salt concentrations of up to 10% NaCl [16], tolerates low-pH environments[16], and grows in a wide range of temperatures (2°C to 45°C) [15] [17]. Moreover, *L*. *monocytogenes* can be persistent in food industry environments where it can survive for several months or years [18–20]. This ability to persist and multiply makes contamination by *L*. *monocytogenes* difficult to control and eradicate. Therefore, food-processing facilities must be designed carefully, with an emphasis on effective cleaning and disinfection operations in the production line.

Various methods are used during cleaning and disinfection processes, both to sanitize food processing environments through the use of alkaline and acid detergents, enzymes and mechanical force on the surface, and to control the organism residing in the food and the processing devices through the use of a wide range of chemical disinfectants as reviewed by Van Houdt and Michiels (2010) [21]. However, contaminated food product recalls involving *L*. *monocytogenes* remain numerous as reported by the U.S. Food and Drug Administration [22–25]. Thus, it is important to find effective ways to prevent the establishment of *L*. *monocytogenes* in production plants and to avoid biofilm formation. Therefore, any factor that affects the early steps of contamination by this pathogen could be exploited, either in itself or in combination with other destructive tools.

Dehydration, drying, or desiccation is often used as a method of preservation of microorganisms as reviewed by Morgan et al (2006) [26]. However, a high survival rate in bacteria after drying is rarely observed, especially without optimization of the drying process (use of protectants, optimal kinetics and temperature). Indeed, fluctuations of relative air humidity (RH) are known to have lethal effects on cells [27–30], but are not well considered as a means to control microorganisms populations. *L*. *monocytogenes* seems to be relatively resistant to desiccation, exhibiting, for example, the capacity to survive 91 days of desiccation on stainless steel at various RHs (2% and 43%) [31]. Another study showed that *L*. *monocytogenes* survived after 30 days of desiccation on stainless steel at 25°C [32]. However, the main parameters known to affect the resistance of microorganisms to dehydration and rehydration have not been sufficiently studied to make conclusions about their efficiency. In particular, the RH level and the kinetics of RH fluctuations could be optimized to improve lethal effect on cells [29, 33]. Indeed, fast changes in RH have, generally, more detrimental effects than progressive ones. Moreover, conditions of low RH, contrary to conditions of high RH, are known to favor the long-term preservation of cells, macromolecular systems, and membranes [34–36].

In this study, viability of four *L*. *monocytogenes* strains (EGDe, CCL500, CCL128, and LO28) was determined after exposure to different conditions of RH fluctuations.

## Materials and Methods

### Bacterial strains and culture conditions

*Listeria monocytogenes* strains were supplied by the Agence nationale de sécurité sanitaire de l’alimentation, de l’environnement et du travail (Anses; Maison-Alfort, France). *Listeria monocytogenes* EGDe strain (BUG1600) (serotype 1/2a) isolated from a case of animal listeriosis [37, 38], CCL500 strain (serotype 1/2b) isolated from milk, CCL128 strain (serotype 4b) isolated from and persistent in the cheese industry, and LO28 strain (serotype 1/2c) isolated from a case of human listeriosis [39], were used in this study. Strains were stored on tryptic soy broth supplemented with 20% glycerol (TSB; Sigma-Aldrich, St. Quentin Fallavier, France) at –80°C. For revitalization, bacteria were isolated with a sterile swab on tryptic soy agar (TSA, Sigma-Aldrich) plate, incubated at 25°C for 48 h and stored at 4°C for a maximal period of one month. Subculture was performed by inoculation of five colonies in 10 mL of TSB and incubated overnight at 25°C. Final culture was performed by diluting the subculture to adjust the final OD_600_ to 0.005 in 50 mL of fresh TSB before incubation at 25°C for 24 h to reach the stationary growth phase.

### Preparation of environmental drying chambers and polypropylene coupon

The controlled environment drying chambers were hermetic plastic boxes (20 cm × 13 cm × 6 cm) containing a saturated salt solution to control RH [29]. A saturated solution of NaCl, KI, K_2_CO_3_, and LiCl (Sigma-Aldrich) was added at the bottom of the drying chambers to obtain 75%, 68%, 43%, and 11% RH in the hermetic box, respectively. A ventilator of 4.1 W (max.) with a diameter of 80 mm and a height of 25 mm comprising seven blades (Sunon, Radiospares, France) was placed in the drying chamber to control the rate of drying.

Coupons were formed from 15 mm × 10 mm × 2 mm rectangles of polypropylene (Scientix, Fougères, France). They were cleaned in 70% ethanol, rinsed with distilled water, and sterilized overnight in an oven at 116°C.

### Dehydration and rehydration processes

#### Dehydration conditions

Stationary growth phase cultures of EGDe, CCL500, CCL128, and LO28 strains (25 mL) were centrifuged for 10 min at 3645 ×*g* and washed once with phosphate-buffered saline (PBS, Sigma-Aldrich). Pellets were suspended in 20 mL of PBS. For desiccation experiments, 10 μL of cell suspension with a final concentration of 1 × 10^9^ CFU/mL was placed onto the hydrophobic polypropylene coupons, a surface that can be found in the food industry in tanks, pipeworks, accessories, and on cutting surfaces [40]. Samples were placed in the drying chambers on a rack to keep them above the salt solutions. The various conditions (11%, 43%, 68%, and 75% RH for 30, 60, 90, 120, and 180 min) were tested while the chambers were maintained at 25°C and ventilated at the same rate to increase desiccation. Variations in the operating rate of the ventilator inside the drying chamber allowed control of the hydric equilibrium between the sample and air with the various rates. The ventilator rate was changed by controlling the input voltage to the ventilator (3, 5, 7.5, and 12 V).

#### Rehydration conditions

For instantaneous rehydration, 1 mL of PBS was deposited on the dried cells which subsequently were detached from the polypropylene coupon by 30 successive aspirating and dispensing cycles using a micropipette. For progressive rehydration, dried cells were introduced into a closed chamber with RH adjusted at 99% for 60 min at 25°C. 1 mL of PBS was then deposited on the wet bacteria and recovered by 30 successive aspirating and dispensing cycles using a micropipette.

In preliminary experiments we checked that no bacteria remained attached to polypropylene coupons after drying and rehydration. Briefly, after recovery of bacteria, coupons were stained for 5 min. with 100 μL of methylene blue (Sigma-Aldrich) solution (0.25 g/L) and rinsed with 1 mL of distilled water. Finally, the absence of bacteria was checked by observation with an optical microscope.

### Dehydration and rehydration cycle

Suspensions of CCL500, EGDe, CCL128, and LO28 strains were prepared as described above. Samples were placed into the drying chambers containing saturated solutions of K_2_CO_3_ or KI reaching 43% and 68% RH, respectively, and the chambers were maintained at 25°C and ventilated for 180 min. At the end of the first cycle, bacterial cells were rehydrated instantaneously with 10 μL of distilled water and samples were reintroduced into the chamber at 25°C for 180 min. At the end of the second cycle, bacterial cells were rehydrated with 1 mL of PBS and were detached from the polypropylene coupons by aspirating and dispensing the samples 30 times using a micropipette.

### Measurement of cell cultivability

The viability of bacteria was estimated by spread plating method. After rehydration, cell suspensions were serially diluted and appropriate 10-fold dilutions were plated on TSA. Colonies were counted after incubation for 48 h at 25°C and recorded as CFU/mL. Viability was expressed as log_10_ (N/N_0_), where N represents the final cell concentration and N_0_ represents the initial cell concentration. The detection threshold of the method representing the maximum loss of detectable viable cell is –4.5.

### Drying kinetics

#### Water loss during desiccation

Four drops of 10 μL of PBS (same volume as the bacterial suspensions) on four polypropylene coupons were weighed with a precision balance (precision ± 0.0003 g, Sartorius, France) after desiccation in a drying chamber at 99%, 75%, 68%, 43%, and 11% RH at 25°C. The weight of the coupons containing the droplets was measured after 1, 5, 10, 15, 30, 45, 60, 90, 120, and 180 min for each condition to obtain curves relating evaporated water mass as a function of drying time.

#### Calculation of drying constants

To characterize the kinetics of drying generated by the different experimental conditions used (RH level and ventilator speed), experimental points were fitted to an exponential curve (Eq 1), i.e. decrease in water mass as a function of time. Using the reduction of the sum of squares scores between the experimental curve and the model (*fminsearch* function in Matlab R2012a), two variables were estimated: τ and *r*. τ is the time constant of the system and represents the time needed to lose 63% of the initial weight. r*0.01 represents the final dried weight present on the coupon.

$$M\left(t\right)=0.01\;(\text{exp}\;\left(\frac{-t}{\tau }\right)+r)$$ (1)

Finally, maximal speed (*V*_max_) for each desiccation at the various relative humidities was calculated as shown in Eq 2.

$$Vmax=\frac{-0.01}{\tau }$$ (2)

### Statistical analysis

All data were collected in four independent experiments. To compare the various results obtained in this study, the variance homogeneity (*F*-test) was tested. Our results revealed a homogeneous variance (*p* > 0.05). An ANOVA and Tukey’s honestly significant difference test (if *p* < 0.05) were performed to determinate whether significant differences existed between different strains. Analyses were performed using R software, version 3.1.2.

## Results

### RH level and drying kinetics affect the survival of *L*. *monocytogenes*

**Fig 1:**
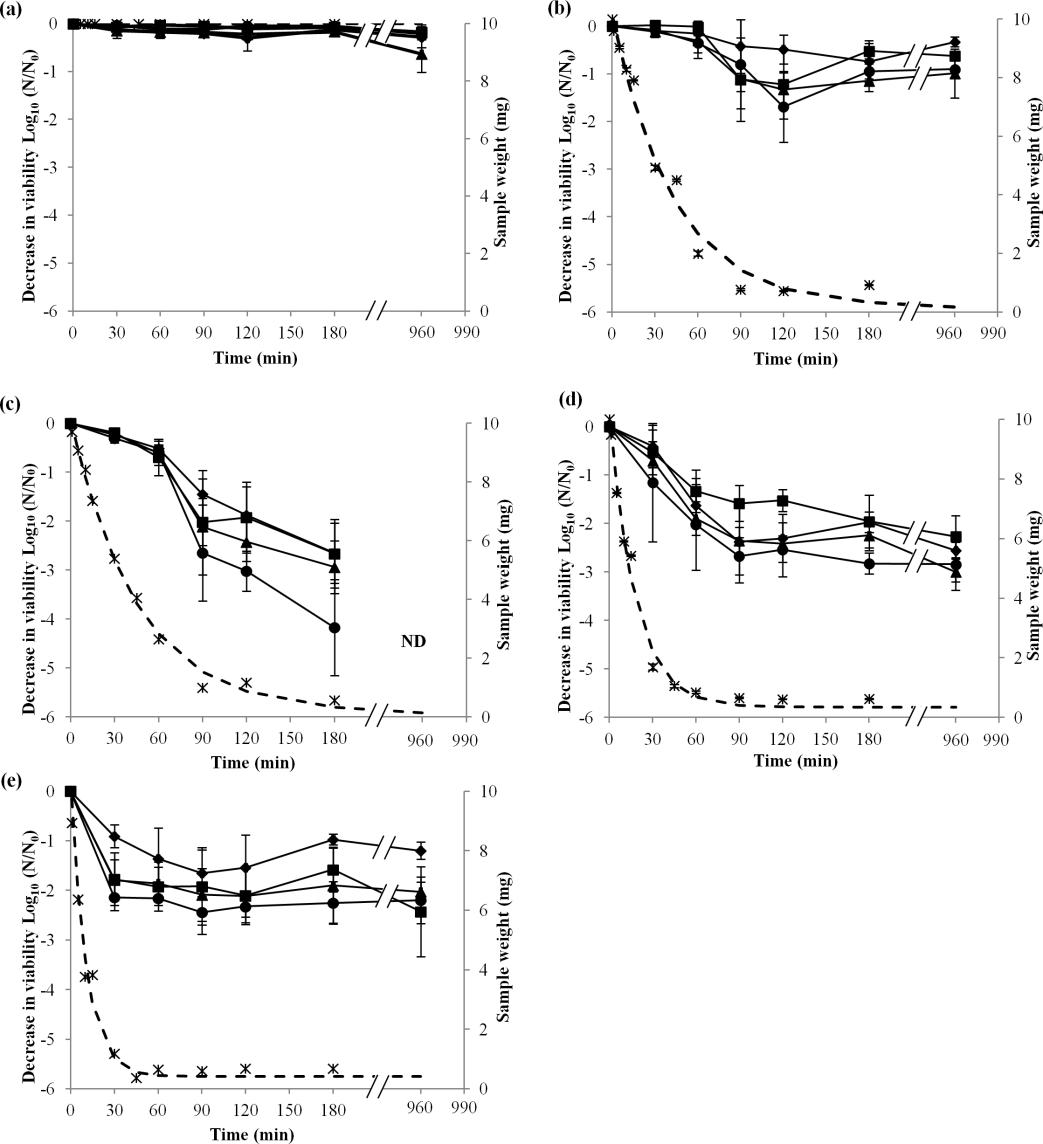
*L*. *monocytogenes* survival at different RH. Decrease in viability of *L*. *monocytogenes* strains, EGDe (square), CCL500 (diamond), CCL128 (triangle), and LO28 (circle), during desiccation on polypropylene coupons incubated at 25°C and 99% RH (a), 75% RH (b), 68% RH (c), 43% RH (d), and 11% RH (e) for 30, 60, 90, 120, 180, and 960 min with an instantaneous rehydration in 1 mL of PBS. Error bars correspond to the SD calculated from four independently repeated experiments. Sample weight was estimated by weighing samples dried at 99% RH, 75% RH, 43% RH, and 11% RH for 1, 5, 10, 15, 30, 45, 60, 90, 120, 180, and 960 min. (ӿ) corresponds to experimental points and (---) corresponds to the curve of the sample weight loss model. ND (not detectable) represents the decrease in viability of any of the strains under the limit of detection by this method.

Viability kinetics of CCL500, LO28, EGDe, and CCL128 *L*. *monocytogenes* strains at 75%, 68%, 43%, and 11% RH in ventilated hermetic boxes for 180 min at 25°C were studied, and in parallel, evaporation of water was measured by weight loss (Fig 1). At 99% RH (control), no evaporation was detected and the viability of the four strains remained stable (Fig 1A). Because ventilator speed was the same for each of the different RH conditions, drying occurred at different rates for the various RHs.

During dehydration at 75% or 68% RH, sample evaporation occurred more slowly than at 43% and 11% RH. Indeed, after 60 min of dehydration, the sample weight was 2 mg at 75% RH, 2.6 mg at 68% RH, 0.85 mg at 43% RH, and 0.65 mg at 11% RH. At 75% and 68% RH, no further weight loss was detected after 90 min of drying. After dehydration at 75% RH, liquid remained on the coupon surface, whereas at 68%, 43%, and 11% RH, a dehydrated cell layer was observed.

Viability of the four strains at 75% RH was stable during the first 60 min of drying and decreased between 60 and 180 min of drying, except for CCL500 viability, which significantly decreased at 180 min of drying (Fig 1B). Between 90 and 180 min of drying, stabilization of viability for CCL500 and CCL128 strains, and a slight, but not significant, increase in the viability of EGDe and LO28 strains were measured. No significant differences were found for each strain, between survival after 180 and 960 min.

Dehydration at 68% RH showed evaporation characteristics almost identical to those at 75% RH. Indeed, at 30 min of drying, sample weight at 68% RH was not significantly different than at 75% RH (Fig 1B and 1C). There was no more weight loss after 90 min of drying. Viability of the four strains of *L*. *monocytogenes* at 68% RH decreased slightly during the first 60 min for all strains. After 60 min, viability continued to decline strongly with a significant difference (*p* < 0.05) to 180 min (Fig 1C). Indeed, at 180 min, there was a significant difference (*p* < 0.05) between the decrease in viability of LO28, which is more sensitive, and the other strains. Cell survival decreased for the four strains between 180 and 960 min after drying at 68% RH, and no bacterial survival was detected at 960 min in any strain, involving a decrease in cell viability of more than 4.5 log_10_ units.

Dehydration at 43% RH was faster than that at 75% and 68% RH. There was no further weight decrease after 90 min of drying (Fig 1D). Viability of the four strains at 43% RH decreased rapidly during the first 90 min of drying. Decrease in viability occurred mainly during water evaporation. Significant differences (*p* < 0.05) between the viability of the four strains dried at 43% RH for 180 min was observed. No significant differences were found for any strains between survival after 180 and 960 min.

Dehydration at 11% RH was faster than that at 43% RH (Fig 1D and 1E). Kinetics of viability at 11% RH were similar to those during drying at 43% RH (Fig 1C), with a decrease in viability during sample evaporation (in this case, 30 min) and a period of stabilization when sample evaporation peaked. No significant differences were found for any strains between survival after 180 and 960 min (Fig 1D).

These experiments revealed that, in all cases, cell death occurred during the transient dehydration step, and thus decrease in viability occurred concomitantly with water evaporation. Cell viability only continued to decline after the transient dehydration step at 68% RH, where hydric equilibrium was reached.

### Influence of drying kinetics on the survival of *L*. *monocytogenes*

As cell death could be influenced by RH level and drying kinetics, we performed additional experiments to study the respective contributions of these linked parameters. To study the effect of RH, two levels of RH (43% and 68%) most lethal to *L*. *monocytogenes* were selected (Fig 1). We performed drying at these RH levels with different drying rates. The various rates were obtained by varying the ventilator rate in the drying chamber. A fast ventilator rate at 68% RH corresponding to a *V*_max_ of 0.38 10^−3^ mg/min and a slow ventilator rate at 43% RH corresponding to a *V*_max_ of 0.32 10^−3^ mg/min had a similar *V*_max_ (Table 1). An increase in ventilator rate allowed modification of the kinetics of drying, allowing equilibrium to be reached more quickly as shown by 3τ values in Table 1. Whatever the drying time considered, results showed that for each strain, no significant influence of drying speed on viability at 180 and 960 min was detected either for 68% RH or 43% RH, except for the EGDe strain to 43% RH at 960 min and LO28 to 68% RH at 180 min. Overall, these results suggested that, in the tested range of drying kinetics, neither duration nor evaporation rate of the transient dehydration step were crucial parameters increasing cell death. The most influential parameter was RH level, and 68% RH was the more detrimental level whatever strain, drying speed, and time considered.

**Table 1:** Drying parameters and viability of *Listeria monocytogenes* dried at 25°C at various dehydration kinetics.

RH drying chambers	Kinetics parameters of dehydration			Decrease in viability of *L*. *monocytogenes* strains (Log_10_ (N/N_0_), (± SD)*)							
	*V*_max_ (10^−3^ mg/min)	τ(min)	Time when 95% of weight sample is evaporated (min) = 3τ	EGDe		CCL500		CCL128		LO28	
				180 min	960 min	180 min	960 min	180 min	960 min	180 min	960 min
43%	0.72	13.69	41.07	–2.32 (± 0.40)^ab^	–3.74 (± 0.6)^c^	–2.59 (± 0.41)^abc^	–3.02 (± 0.28)^abc^	–2.77 (± 0.29)^ab^	–3.49 (± 0.24)^b^	–3.11 (± 0.17)^a^	–3.97 (± 0.26)^a^
	0.56°	17.81	53.43	–1.95 (± 0.54)^a^	–2.27 (± 0.43)^ab^	–1.97 (± 0.21)^a^	–2.55 (± 0.16)^abc^	–2.24 (± 0.31)^a^	–3 (± 0.2)^ab^	–2.82 (± 0.21)^a^	–2.84 (± 0.52)^a^
	0.32	31.24	93.72	–2.43 (± 0.78)^ab^	–3.36 ± (0.65)^bc^	–2.36 (± 0.72)^ab^	–3.1 (±0.35)^bc^	–2.51 (± 0.55)^ab^	–3.48 (±0.35)^b^	–3.22 (± 0.31)^a^	–4.1 (± 0.54)^a^
68%	0.38	25.8	77.4	–3.85 (± 0.07)^c^	< –4.5^d^	–3.63 (± 0.32)^c^	< –4.5^d^	–4.15 (± 0.36)^b^	< –4.5^c^	< –4.5^b^	< –4.5^b^
	0.28	34.9	104.7	–3.08 (± 0.38)^abc^	< –4.5^d^	–2.81 (± 0.76)^abc^	< –4.5^d^	–3.47 (± 0.94)^b^	< –4.5^c^	–3.82(± 1.11)^a^	< –4.5^b^
	0.22°	45.71	137.13	–2.66 (± 0.7)^abc^	< –4.5^d^	–2.65 (± 0.61)^abc^	< –4.5^d^	–2.93 (± 0.53)^ab^	< –4.5^c^	–4.17 (± 0.98)^a^	< –4.5^b^

Values for each strain followed by different letters indicate significant differences (p < 0.05) as determined by the Tukey post hoc test.

° Results correspond to conditions studied in Fig 1.

### Impact of rehydration kinetics on *L*. *monocytogenes* survival

**Fig 2:**
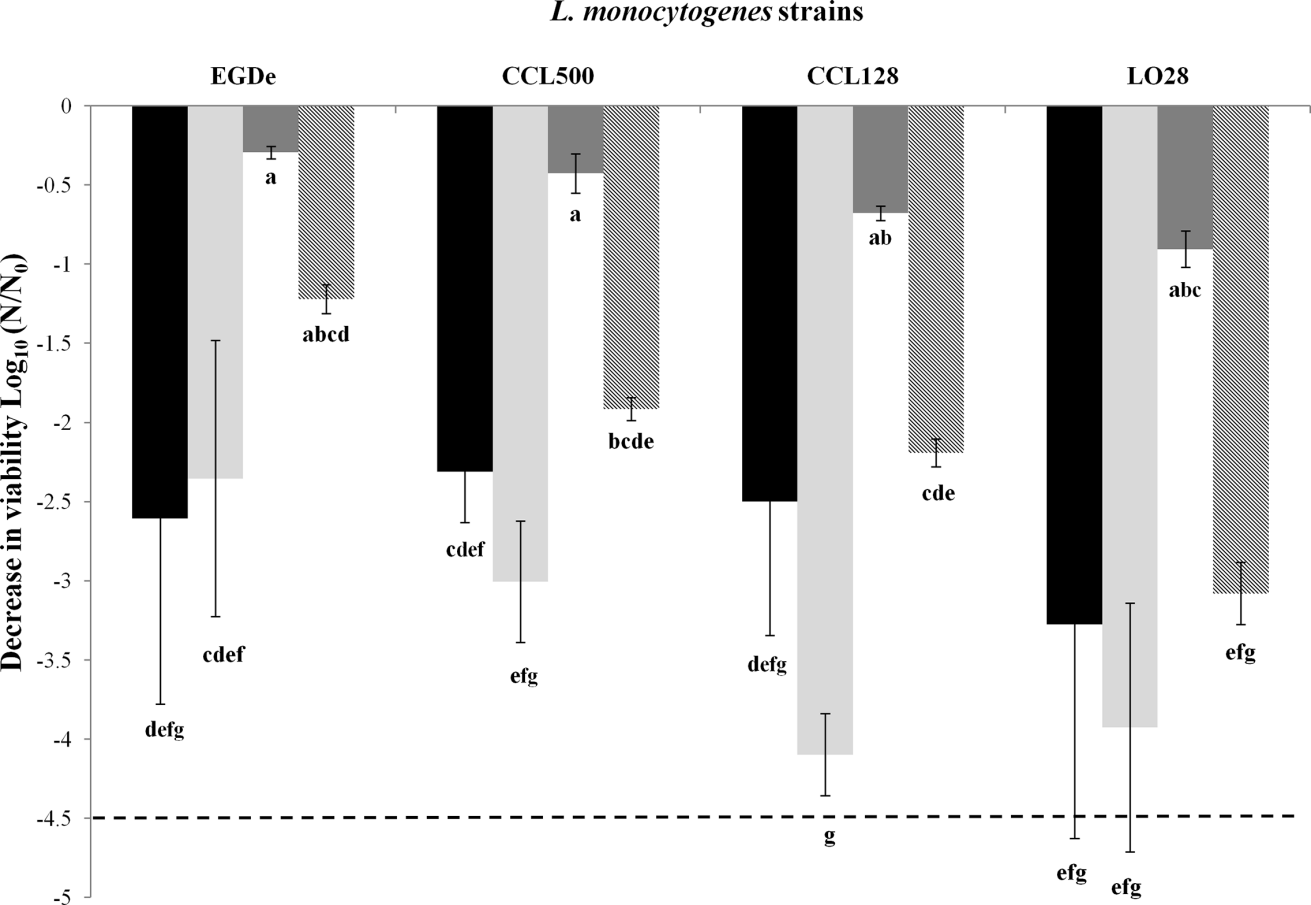
*L*. *monocytogenes* survival after different kinetics of rehydration. Decrease in viability of *L*. *monocytogenes* strains (EGDe, CCL500, CCL128, and LO28 strains) in PBS during desiccation on polypropylene coupons incubated at 25°C and at 43% RH or 68% RH for 180 min. Initial concentration was equivalent to 10^9^ CFU/mL. Two models of rehydration were studied: an instantaneous rehydration and a progressive rehydration. Black bars correspond to drying at 68% RH with a slow rehydration and light gray bars correspond to drying at 68% RH with a rapid rehydration. Dark gray bars correspond to drying at 43% RH with a slow rehydration and hatched bars correspond to drying at 43% RH with a rapid rehydration. Error bars correspond to the SD calculated from four repeated experiments. (---) corresponds to the method detection limit.

We assumed that rehydration conditions could play a role in cell death, regardless of drying ones [29, 33]. To study the impact of this parameter, dried samples were rehydrated instantaneously by direct immersion into PBS or progressively by equilibration in an atmosphere with high RH (99%). These experiments were conducted at a RH of 43% or 68% (Fig 1). Results are reported in Fig 2, and taking into account rehydration kinetics and strains, ANOVA showed a significant difference (*p* < 0.05) in decrease in viability for each strain and according to the rehydration kinetics. Cell survival was markedly influenced by the rehydration kinetics (except for EGDe); instantaneous rehydration was more detrimental to cells than progressive rehydration. However, drying at 68% RH for 180 min followed by progressive and instantaneous rehydration did not significantly influence rehydration kinetics in any strain.

### Effect of an additional dehydration–rehydration cycle

Because the main objective of this study was to check the efficiency of atmospheric RH fluctuations for *L*. *monocytogenes* decontamination, we tried to improve the lethal effect by applying two consecutive dehydration–rehydration cycles. For this purpose, cells were equilibrated at 43% and 68% RH for 180 min per cycle with instantaneous rehydration.

**Fig 3:**
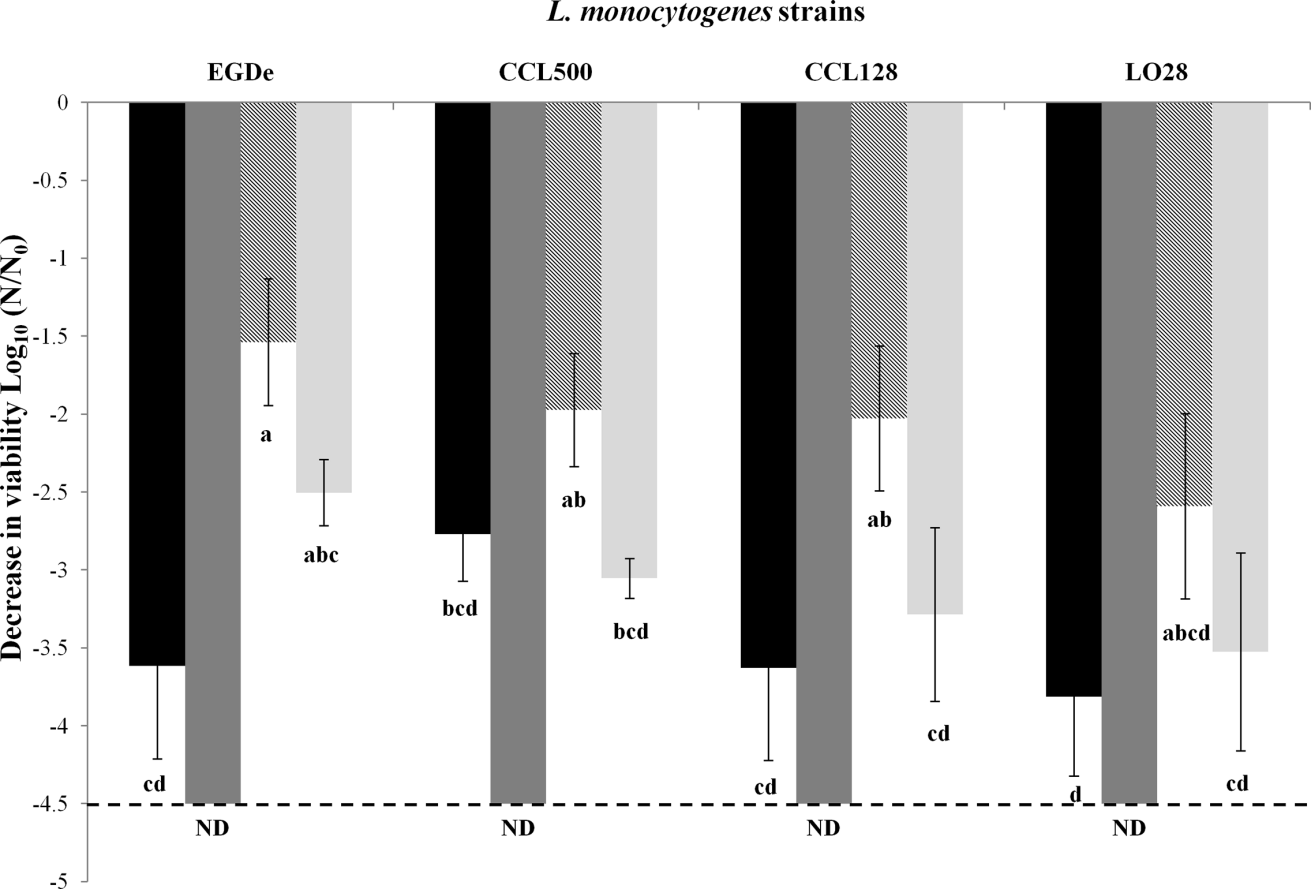
Impact of dehydration-rehydration cycle on *L*. *monocytogenes* survival. Decrease in viability of *L*. *monocytogenes* EGDe, CCL500, CCL128 and LO28 strains in PBS during desiccation at 43% RH and 68% RH on polypropylene coupons with one cycle at 25°C for 180 min or with two successive cycles at 25°C for 180 min each with an instantaneous rehydration in 10 μL of distilled water between the two cycles. Initial concentration was equivalent to 10^9^ CFU/mL. Black bars correspond to drying at 68% RH for one cycle and dark gray bars correspond to drying at 68% RH for two cycles. Hatched bars correspond to drying at 43% RH for one cycle and light gray bars correspond to drying at 43% RH for two cycles. Error bars correspond to the SD calculated from four repeated experiments. (---) corresponds to the method detection limit. ND (not detectable) represents decrease in viability of fours strains which is under method detection limit.

For all strains, after two consecutive dehydration–rehydration cycles, a greater decrease in viability was observed than after only one cycle (Fig 3). ANOVA showed a significant difference in decrease in viability between the four strains among the different treatments. No bacterial viability was detected after the application of two dehydration–rehydration cycles to 68% RH for each strain (Fig 3). Therefore, an additional dehydration–rehydration cycle significantly increased the decrease in viability.

## Discussion

Results of our study showed that ambient RH fluctuations are an efficient means by which to kill *L*. *monocytogenes* freshly deposited onto an inert surface. In particular, cell death is promoted by a transient dehydration step followed by instantaneous rehydration. Cell death is also favored by maintaining cells at 68% RH; in this particular case, mode of rehydration did not influence cultivability. To explain these lethal effects, the main mechanisms causing cell degradation during dehydration–rehydration or desiccation–wetting need to be considered.

### Causes of death of *L*. *monocytogenes* during hydric fluctuations

From a physicochemical point of view, cell death under severe dehydration–rehydration is generally related to three main constraints that are mechanical, structural and oxidative [41, 42]. The mechanical constraint applied to cells results from mass transfers between cells and their surrounding environments; it consists mainly of osmotic flows, which cause a cell volume decrease during dehydration and then swelling during rehydration[43]. A direct consequence of osmotic stress is an alteration of the cell plasma membrane during dehydration, with reported formation of invaginations and eventually formation of plasma membrane vesicles that lead to a decrease in the membrane area [33, 44]. Instantaneous rehydration induces fast water re-entry and leads to cell lysis as observed in bacteria and yeast; by contrast, progressive rehydration favors membrane integrity recovery. These mechanical events could actually explain results presented in our study because cell death was mainly related to the transient drying step (Fig 1) and because instantaneous rehydration promoted it (Fig 2). Therefore, mechanical stress could contribute to the lethal effect observed and could explain why increase desiccation tolerance was observed for *L*. *monocytogenes* presenting a putatively less fluid membrane [45]. Oxidation is also proposed to explain cell death during desiccation of *L*. *monocytogenes* [45]. Oxidation is thought to result from cell metabolic dysfunction and arrest caused by dehydration [42]. Accordingly, most reactive oxygen species that are generated by cellular reactions are supported by enzymatic systems that are denatured by dehydration. Oxidation is also related to the transition from the liquid to the aerial medium, which increases the direct contact between samples and gaseous oxygen (oxygen that is less concentrated and less mobile in water than in air). In the case of our experiments, this last phenomenon occurs below 75% RH. Accordingly, the bacterial suspension medium (PBS) is mainly composed of NaCl, and this salt solution displays a water activity of 0.75 at 25°C in saturated solution [46]. This means that exposure of PBS to 75% RH induces water evaporation from the sample until the formation of a saturated liquid solution (crystal formation) of NaCl. This was confirmed by our experiments because bacterial samples exposed to 75% RH remained liquid at the end of the dehydration process. In these conditions, cells were exposed to increased osmotic pressure (and therefore to mechanical and structural constraints) without direct contact with air. This may be the reason that treatment at 75% RH was less detrimental to cells than at other RH levels tested (Fig 1). Nevertheless, survival observed at 68% RH cannot result exclusively from this phenomenon, because lethality is stabilized after transient steps of drying to 43% and 11% RH (Fig 1). Increased mortality and its progression during the holding time at 68% RH could also be related to any impact of drying level on molecular mobility. This phenomenon is exploited in many preservation methods, where biological samples are extensively dehydrated to ensure their long-term preservation. Typically, freeze-dried bacteria are preserved at below 15% RH and their preservation period is considerably reduced if this value is increased [36]. This phenomenon is related to molecular mobility, which is decreased by a few orders of magnitude when water is drastically removed [41]. Therefore, even if cell structures are disorganized or radical species are present, their displacements and interactions leading to reactions or oxidation are dramatically slowed during holding time in very dry conditions. This may explain the decrease of *L*. *monocytogenes* viability during its maintenance at 68% RH. Indeed, this relative humidity is low enough to promote mechanical, structural, and oxidative damages, but high enough to maintain the mobility of the cell constituents.

In our study, survival rates were generally low if compared to other studies. For example, drying the Lm568 strain with 43% RH induced a decrease in viability of –1.48 log_10_ on the first day of desiccation [45], while our study showed a decrease in viability from 2 to 4 log_10_ according to the strain studied (Table 1). Indeed, the survival rate obtained in our study was inferior, but the difference from the study of Hingston et al. (2015) could be explained by the fact that the stress response is strain dependent or by differences between the drying mediums. In our study, bacteria were dried in PBS (nutrient-free medium), while in the study of Hingston et al. (2015), bacteria were dried in nutrient medium (TSB). In PBS, bacteria may lack sufficient nutrients and energy to set up a stress response related to active mechanisms (energy production, membrane transport, protein metabolism, lipid biosynthesis, and oxidative damage). The low viability rate obtained in this study indicates that active compensation of cellular damage did not induce or was not sufficient to compensate for the physicochemical consequences of dehydration under our experimental conditions.

### Efficiency of RH regulation in improving bacterial decontamination on surfaces

Finally, the main objective of this study was to evaluate the potential use of RH variation as a tool for the destruction of freshly deposited bacteria to avoid their growth on inert surfaces. We showed that this strategy is efficient and that optimal conditions can be selected. We found that the optimal conditions are an ambient RH of 68%. This level of RH promotes cell destruction during transient drying steps and promotes continuous cell destruction while they are dried (Fig 1 and Table 1), whatever mode of rehydration is applied (Fig 2). Other levels of RH are less efficient and require instantaneous rehydration or supplementary cycles of dehydration–rehydration. A level of 68% RH has the advantage of not being excessively low, because low RH levels are difficult to achieve in food plants where water is omnipresent. It is important to note that RH levels held for long periods between 50% and 70% RH are known to be detrimental to the preservation of many dried microorganisms [28]. Here we studied only *L*. *monocytogenes*, known for its high resistance to hydric stress [47, 48]. Our results provide good leads for the development of industrial strategies. Indeed, it will be interesting to effectively apply RH as a tool to control foodborne pathogens in the food industry, reducing the requirements for disinfectants and biocides, which are harmful to the environment.

## Conclusions

This study about the impact of RH variation on the survival of *L*. *monocytogenes* contaminating clean surfaces revealed that RH offers the potential to reduce the establishment of pathogens in industrial premises. From our results, we conclude that the main principle involved is a decrease in RH to a level that is not so low that it stabilizes cells, but sufficiently high to induce dehydration-related damage. By contrast with temperature and chemical agents, largely used as technological environmental parameters for the effective control of microorganism activities in rooms or plants, RH was not considered as an effective means by which to increase the safety of industrial environments. This study shows that RH control might offer new optimization ways to reduce bacterial contamination in food production plants and other environments, such as hospitals, schools, or conveyances. Important research remains to be conducted before the application of RH for control of bacterial contamination can be introduced. A study of the contribution of other parameters, to observe the lethal effects of RH fluctuation, is necessary. These parameters include resistance of spoilage and other pathogenic microorganisms (bacteria, yeast, and molds), the influence of substrates (from meat, fish, and juices) in the suspension medium before dehydration, and other surface materials. Finally, we plan to perform industrial assays under field conditions.
